# Multiple concurrent and convergent stages of genome reduction in bacterial symbionts across a stink bug family

**DOI:** 10.1038/s41598-021-86574-8

**Published:** 2021-04-08

**Authors:** Alejandro Otero-Bravo, Zakee L. Sabree

**Affiliations:** 1 Department of Evolution, Ecology and Organismal Biology, Ohio State University, 318 W. 12th Avenue, Columbus, OH 43210 USA; 2 Nationwide Children’s Hospital, Columbus, OH 43205 USA

**Keywords:** Molecular evolution, Bacterial evolution, Symbiosis

## Abstract

Nutritional symbioses between bacteria and insects are prevalent and diverse, allowing insects to expand their feeding strategies and niches. A common consequence of long-term associations is a considerable reduction in symbiont genome size likely influenced by the radical shift in selective pressures as a result of the less variable environment within the host. While several of these cases can be found across distinct insect species, most examples provide a limited view of a single or few stages of the process of genome reduction. Stink bugs (Pentatomidae) contain inherited gamma-proteobacterial symbionts in a modified organ in their midgut and are an example of a long-term nutritional symbiosis, but multiple cases of new symbiont acquisition throughout the history of the family have been described. We sequenced the genomes of 11 symbionts of stink bugs with sizes that ranged from equal to those of their free-living relatives to less than 20%. Comparative genomics of these and previously sequenced symbionts revealed initial stages of genome reduction including an initial pseudogenization before genome reduction, followed by multiple stages of progressive degeneration of existing metabolic pathways likely to impact host interactions such as cell wall component biosynthesis. Amino acid biosynthesis pathways were retained in a similar manner as in other nutritional symbionts. Stink bug symbionts display convergent genome reduction events showing progressive changes from a free-living bacterium to a host-dependent symbiont. This system can therefore be used to study convergent genome evolution of symbiosis at a scale not previously available.

## Introduction

Animal-microbe associations are prevalent throughout the tree of life and can greatly benefit and expand the host’s ability to survive in a variety of environments. Hosts can assimilate resources produced by their bacterial symbionts, thereby benefitting from entire metabolic pathways that they are unlikely to develop^1^. Insects, particularly hemipterans, have used these symbioses to expand their feeding strategies to include nutrient-poor or unbalanced resources^2^. Resource provisioning-based mutualisms between insects and bacteria have been well-documented in many insects, including aphids^3^, cockroaches^4^, armored scales^5^, cotton stainers^6^. Bacterial partners of insects can be members of diverse phyla, including Proteobacteria and Bacteroidetes, and comparative genomic analyses reveal that they functionally converge around the supplementation of essential amino acids and/or vitamins absent from the host’s diet^7^. Host-microbial mutualisms often involve modifications to the involved partners that are evident at genomic, transcriptomic, and physiological scales, and some common symptoms include the evolution of specialized host cells and organs to house symbionts^8–11^ and genomic streamlining in the symbiont^7,12^.

Some patterns of genomic modifications in bacterial symbionts have emerged across a wide range of symbioses. When these symbiont genomes are compared to those of free-living close relatives, the evolutionary pressures that may be unique to endosymbiotic lifestyles come into focus^13^. Dramatic gene losses are often observed in bacterial symbionts and this phenomenon is thought to be due, in part, to relatively stable environmental conditions within host tissues. Genome shrinkage is due primarily to relaxed selective pressures upon genes not essential for survival in the host and genetic bottlenecks resulting from vertical transmission^14^. For example, genes involved in cell wall biosynthesis and self-defense are consistently absent from symbiont genomes^15^. As a result, core symbiont genomes are consistently reduced relative to their free-living close relatives^9^. Pseudogenization of genes experiencing relaxed selection often precedes their loss^16^, however it is rare to find bacterial-insect symbioses within a single insect family undergoing radically different stages of gene loss, which would greatly facilitate the study of this process.

Members of the Pentatomomorpha often harbor an extracellular bacterial symbiont within posterior midgut crypts^17–19^. While this symbiotic organ is fairly similar across a wide variety of families, other traits of the symbiosis can be quite variable, including the acquisition strategy^17,20–23^, degree of cophylogeny between host and symbiont^24^, and the symbiont’s reliance on its host^17^. In the Pentatomidae, symbionts are typically vertically-inherited by nymphal consumption of maternal deposits of symbiont-enriched secretions during emergence from eggs. While symbionts can vary between cultivable and uncultivable, they are nonetheless transmitted extracellularly, unlike intracellularly-transmitted symbionts in other insects^17,23,25^. Some pentatomids can harbor both vertically and environmentally-acquired symbionts depending on which bacteria they were exposed to during their first instar^26^. For example, *Plautia stali* (Pentatomidae) can domicile up to six different symbiont ‘types’ (named A through F), that vary in origin and degree of host reliance^27^. However, the most common scenario is that of more specific host-symbiont associations, where a single host species is consistently associated with a single symbiont^24,28–31^, and cophylogeny is detected between genera^29,32,33^ but not necessarily at higher taxonomic levels^34,35^, which would indicate multiple associations at different points in time throughout the family.

Since the Pentatomidae have a conserved symbiotic organ but an imperfect and variable symbiont transmission mechanism and multiple symbiont acquisitions, different taxonomically distant species are likely to contain symbionts at different stages of the symbiosis. Previously, two cases of genome reduced symbionts in two distinct subfamilies within the Pentatomidae were identified with different degrees of genome reduction that originated separately^35–37^. This was identified as a potentially valuable system to understand the convergent evolution of nutritional symbioses from the bacterial symbiont’s perspective^18^. Therefore, sampling of known symbionts in this family by genome sequencing will shed light into the diversity and variability of these symbionts. A wide range of genome sizes across different species of the Pentatomidae were observed and the core genome of these symbionts and metabolic pathways were predicted to be retained or lost at different stages of genome shrinkage.

## Methods

### Genome sequencing and assembly

Collection permits for stink bugs were obtained from Costa Rican authorities (MINAET). Wild-caught stink bugs belonging to the subfamily Edessinae and Pentatominae were collected at La Selva Biological Station, Organization for Tropical Studies (coordinates 10.43028535, -84.007099) during June, 2017. These included *Edessa oxcarti*, *E*. sp, and *Brachystethus rubromaculatus* for the Edessinae and *Arvelius albopunctatus, Sibaria englemani*, *Taurocerus edessoides*, *Mormidea* sp*.,* and an unidentified Pentatomidae species for the Pentatomidae. Additionally, individuals of the Brown Stink Bug *Euschistus servus* and Green Stink Bug *Nezara viridula* were donated by Michael Toews (from Tifton, GA) and individuals of the Harlequin Bug *Murgantia histrionica* were donated by Don Weber (from Beltsville, MD). Morphological identification was carried out with appropriate guides^38–44^ and confirmed by molecular analysis as described below. DNA extraction and genome sequencing were performed as in^36^. Briefly, insects were surface-sterilized with three washes of ethanol 70% and the symbiotic organ was dissected. DNA was extracted from the whole organ of individual specimens using the DNeasy Blood and Tissue kit (Qiagen) with RNAse treatment according to manufacturer instructions. Paired 300 bp Nextera Libraries were prepared and sequenced using Illumina MiSeq at the Ohio State MCIC with read lengths of 2 × 300 bp. Raw reads were corrected using Trimmomatic^45^ and assembly was performed using Unicycler 0.4.8^46^. Resulting contigs were filtered based on coverage level and scaffold linkage using Bandage 0.8.0^47^. Additionally, BLASTn^48^ searches were employed to separate host and symbiont sequences. Host mitochondria was obtained by searching resulting contigs for the 13 genes of stink bug mitochondria or performing de-novo assembly on a subset of reads obtained by mapping to these contigs using BWA^49^ and SAMtools^50^ until recovering a circularized mitochondrial genome.

### Annotation and genome comparisons

Genomes were annotated using Prokka^51^, RAST^52^, and the NCBI’s PGAP^53,54^, and annotations were visualized using Geneious 8.1.9 (https://www.geneious.com). Genome completeness was assessed with Benchmarking Universal Single Copy Orthologs (BUSCO) 2.0.1^55^ which compares gene content with a single copy ortholog set specific for gammaproteobacteria. Host mitochondria were annotated using the Geneious ‘Annotate’ feature with a database of available Pentatomomorpha mitochondria and manually curated. For analyses including shared gene content, Roary 3.11.0^56^ was used on previously sequenced stink bug symbiont genomes and representative genomes of species of the genus *Pantoea.* Functional protein-coding gene categorization and completeness of metabolic pathways were determined by referring to KAAS^57^, eggNOG 4.5.1^58^ and MetaCyc databases^59^.

### Symbiont identification

It has been well documented that genome reduced symbionts are difficult to accurately place in a phylogeny due to their high mutation rates creating long branch attraction^60^, which can lead to erroneous predictions in tree-based identification methods. Additionally, this fast mutation rate significantly decreases the sequence identity between genome reduced symbionts and other bacteria. In order to identify sequenced genomes we used SINA^61^ which uses ribosomal RNA genes to classify sequences with the Least Common Ancestor method using available sequences. Additionally, we used a modified method for estimating a phylogenetic tree for these species as described in^36^ in which we estimated separate trees for each genome reduced bacteria with the other non-genome reduced members as well as members of the Erwiniaceae (Table S2) using FastTree 2.1.11^62^, which would prevent some of the long branch attraction artifacts, and evaluated the consensus of all trees. Alignments were produced from the 10 largest protein coding genes common to all strains by reciprocal best hit BLAST and aligned using TranslatorX^63^.

### Host mitochondrial phylogeny

Cytochrome oxidase I (COX1) genes from the mitochondrial genomes sequenced were searched against the BOLD database^64^ to corroborate morphological identification (Table S1). Additionally, all 13 coding regions and two rRNA regions were aligned with MUSCLE^65^ using the default parameters. Protein sequences were manually inspected and corrected using the translation alignment feature from Geneious for the recovered mitochondrial genomes as well as for other complete pentatomid mitochondria. *Coptosoma bifaria* (Pentatomoidea; Plataspidae, NCBI accession number EU427334.1) was used as the outgroup. Trees were reconstructed using RAxML^66^ with separate partitions for each codon and the rDNA genes, and MrBayes^67^ with 4 heated chains and 10,000,000 iterations, with burn-in of 1,000,000 and sampling every 1,000.

## Results

### Genome assembly

Sequencing resulted in libraries containing between 8 and 12 million paired reads for the different samples. After removing adapters and low-quality bases, individual genome assemblies were performed and each resulted in few-to-many scaffolds (ranging between 9 and 285 per assembly, see Table 1), forming a single connected component of ambiguously connected scaffolds ranging from 0.82 to 5.6 Mb with few or no dead ends. Separately, between one and three contigs belonging to the host mitochondria or circular high coverage plasmids were found. Scaffolds of all the main components had BLAST matches to bacteria from the Enterobacteriaceae, namely *Pantoea*, *Erwinia*, and other genome-reduced insect symbionts. Circularizing the genomes was not possible using only the Illumina data due to most components converging multiple times on contigs containing the 5S, 16S, and 23S rRNA genes, which are repeated across the genome. Despite this, we are confident that the genomes described represent the complete gene set for each symbiont with few and negligible omissions given that: 1) the assemblies contained no additional contigs of similar or higher coverage, 2) the evidence that each contig was connected to the main component via the ribosomal gene operon (with the exception of circular plasmids) which is present multiple times in each genome, 3) the multiple assemblies for symbionts of the *Edessa* matched in contig number and connections to the repetitive sequences, and 4) in the case of the unreduced genomes, the BUSCO completeness was above 96%.

**Table 1:** Bacterial symbiont genomes of the Pentatomidae. Genome characteristics were derived from the corresponding genomes deposited in public databases. Mean cov.: mean x-fold coverage of genome sequence as assessed by Geneious.

Name	SILVA Classification	Host	Subfamily	Genome Size (Mb)	Busco Completeness (%)	G + C%	Coding density (%)	Genes	Mean Cov	Scaffolds	References
SoEE	Enterobacterales	*Edessa eburatula*	Edessinae	0.85	73.4	26.9	84.3	750	86	12	Otero-Bravo and Sabree^29^
SoEL	Enterobacterales	*Edessa loxdalii*	Edessinae	0.837	73	27.6	84.3	741	96	12	Otero-Bravo and Sabree^29^
SoEO	Enterobacterales	*Edessa bella*	Edessinae	0.836	71.6	26.6	81.6	721	206	12	Otero-Bravo and Sabree^29^
SoET	Enterobacterales	*Edessa sp. 2*	Edessinae	0.841	70.7	27.8	82.7	733	172	11	Otero-Bravo and Sabree^29^
SoEOx	Enterobacterales	*Edessa oxcartii*	Edessinae	0.827	71.7	26.8	84.4	731	940	12	This paper
SoEF	Enterobacterales	*Edessa F*	Edessinae	0.839	69.5	27.8	82.1	739	860	21	This paper
SoBr	Enterobacterales	*Brachystethus rubromaculatus*	Edessinae	0.843	69.9	25.5	80.7	761	430	30	This paper
SoMh	*Pantoea*	*Murgantia histrionica*	Pentatominae	1.025	75.9	27.1	70.8	794	110	17	This paper
SoAa	Erwiniaceae	*Arvelius albopunctatus*	Pentatominae	1.143	81.4	27.9	77.5	943	190	9	This paper
SoNv	*Pantoea*	*Nezara viridula*	Pentatominae	1.425	87.2	40.6	68.9	1045	70	11	This paper
SoEus	*Pantoea*	*Euschistus servus*	Pentatominae	3.928	96.7	55.2	81.8	3998	25	30	This paper
SoPt	*Pantoea*	*Pentatomidae sp.*	Pentatominae	4.314	98.9	57.7	86.7	4142	79	102	This paper
SoSe	*Pantoea*	*Sibaria englemanni*	Pentatominae	5.046	96.7	53.9	88.3	4764	5	49	This paper
SoTe	*Pantoea*	*Taurocerus edessoides*	Pentatominae	5.458	100	53.7	87.7	5099	35	70	This paper
SoMo	*Pantoea*	*Mormidea sp.*	Pentatominae	5.612	99.1	56.9	85	5497	5.5	285	This paper
*Can* P. carbekii	*Can ‘*P. carbekii’	*Halyomorpha halys*	Pentatominae	1.151	75.8	30.4	69.1	877	71	1	Kenyon et al.^37^
SoPS-A	*Pantoea*	*Plautia stali*	Pentatominae	4.093	97.8	57	84.8	5202	20.5	131	Hosokawa et al.^35^
SoPS-B	*Pantoea*	*Plautia stali*	Pentatominae	2.429	92.9	55.9	72.5	3214	89	34	Hosokawa et al.^35^
SoPS-C	*Pantoea*	*Plautia stali*	Pentatominae	5.138	99.1	57.4	86.7	4850	18	201	Hosokawa et al.^35^
SoPS-D	*Pantoea*	*Plautia stali*	Pentatominae	5.531	97.1	53.8	86.4	5299	19	235	Hosokawa et al.^35^
SoPS-E	*Pantoea*	*Plautia stali*	Pentatominae	5.406	98	53.7	87.2	5135	19	146	Hosokawa et al.^35^
SoPS-F	*Pantoea*	*Plautia stali*	Pentatominae	4.665	98.9	56.7	86.2	4381	23	103	Hosokawa et al.^35^

### Symbiont genome annotation

The genomes of symbionts of stink bugs belonging to the Edessinae and Pentatominae subfamilies were sequenced and annotated. Symbionts of Edessinae stinkbugs, which included the symbionts of six *Edessa* species (hereafter called SoE) and the symbiont of *Brachystethus rubromaculatus* (henceforth SoBr), had genomes that were < 1 Mb in size, varying little in size, and taxonomically categorized by SINA as ‘unclassified Enterobacteriaceae’ (See Table 1, S3). Symbionts of the Pentatominae stink bugs were larger than 1 Mb, ranging from 1 to 5.6 Mb, and were assigned to the *Pantoea* genus.

The smallest genomes among the symbionts of the Pentatominae belong to the symbiont of the harlequin bug *Murgantia histrionica* (henceforth SoMh) at 1.02 Mb, followed by the symbiont of *Arvelius albopunctatus* (henceforth SoAa) at 1.14 Mb, and the symbiont of the brown marmorated stink bug (*Halyomorpha halys*)*, Candidatus* ‘Pantoea carbekii’ (Kenyon, et al., 2014) (henceforth *P. carbekii*) at 1.15 Mb. The symbiont of the green stink Bug, *Nezara viridula* (henceforth SoNv) showed a slightly larger size, at 1.42 Mb. This is followed by a much larger genome, belonging to the B-type symbiont of *Plautia stali* (henceforth SoPs-B) with the smallest symbiont genome identified for this host at 2.4 Mb. Finally, the remaining symbionts contained genomes larger than 3.9 Mb, which is within the range of non-stink bug associated strains of the *Pantoea* genus.

**Figure 1:**
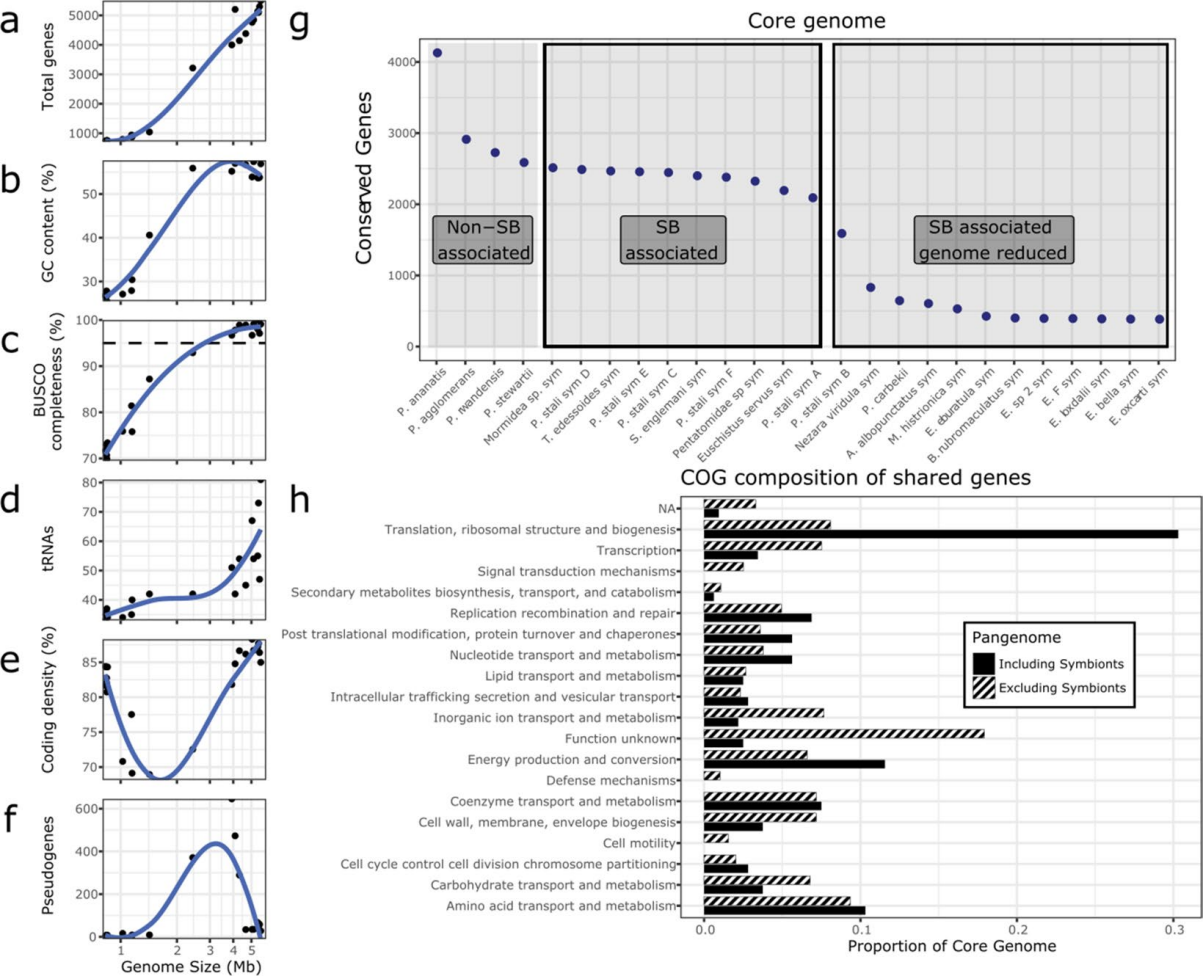
Genomic characteristics of pentatomid symbionts. (**a**–**f**) Genomic characteristics as a function of genome size for stink bug symbionts. (**a**) number of genes annotated, (**b**) GC % of the entire genome, (**c**) percentage of conserved genes found according to the BUSCO gammaproteobacterial set (Dotted line in indicates the recommended 95% threshold of BUSCO completeness for new species descriptions). (**d**) tRNAs annotated, (**e**) coding density of the genome, (**f**) number of pseudogenes annotated by Prokka. Blue line indicates the LOWESS fitted curve. (**g**) Number of conserved genes between each genome and all larger genomes (excluding pseudogenes). (**h**) COG composition of the shared genes for non-symbiotic and large genome symbionts (> 3.8 Mb, dashed bar) and for all genomes including genome reduced symbionts (< 1.4 Mb, solid bar).

Metrics such as gene number, GC content, and genome completeness (Table 1, Fig. 1a-c) were also in accordance to expectations for genomes of given size: GC content showed little variation from the range between 53 and 57% among unreduced genomes and even the moderately reduced genome of SoPs-B (at 2.4 Mb). Reduced genomes on the other hand, presented a GC bias characteristic of genome reduction, ranging from 25 to 30%, while the intermediately sized SoNv showed a slightly lesser skew, with a GC content of 40% (Table 1, Fig. 1b). BUSCO completeness and number of total genes also decreased with greater genome reduction, with intermediate genomes of SoPs-B and SoNv also having intermediate values (Table 1, Fig. 1c,d). Coding density and pseudogene number followed a different trend, where both large and small genomes showed similar values (near 100% and 0, respectively), while intermediate sized genomes deviated significantly from this (Fig. 1e,f).

The subset of genes that is common to all strains, was obtained for the four species of the *Pantoea* genus with complete representative genomes available (which are not associated with stink bugs) and the symbiont genomes used for previous analyses (Table 1). The number of shared genes plateaus for all genomes larger than 4 Mb, following the trend from the non-SB associated strains. This subset of approximately 2450 genes likely represents the core genome of the genus *Pantoea*, which most stink bug symbionts have been assigned to^68^. However, the number of shared genes begins decreasing with the addition of the symbiont of *Euschistus servus* (henceforth SoEus) which despite a relatively large 3.9 Mb genome has lost some of these conserved genes due to pseudogenization. Subsequently, the number of shared genes rapidly decays with the genomes of SoPs-B (2.4 Mb) and SoNv (1.4 Mb). The reduction between the next few genomes is modest due to their similar size of 1 Mb, followed by a larger decrease when the most reduced genomes (< 1 Mb) are added. The set of shared genes for the studied genomes, including all symbionts and non-stink bug associated *Pantoea*, includes 454 genes (Fig. 1g).

The differences between the two sets of shared genes can yield insights into the change in requirements for the symbiotic lifestyle (Fig. 1h). Primarily, the proportion of genes in the translation, ribosomal structure and biogenesis (J) and energy production (C) is much higher for the shared gene set including symbionts than the set excluding them. This difference comes at the expense of a large loss of proteins with poorly characterized function, in the category unknown function (S), general function prediction only (R), or no COG category assigned. Notably, the categories cell motility (N), defense mechanisms (V), and signal transduction mechanisms (T) contain genes shared between the large genomes, but are completely absent when including reduced genomes.

### Host mitochondrial genome sequences and identification

Host mitochondrial scaffolds were recovered from the assembly as a single circular chromosome or in some cases two or three scaffolds that would map to the other stink bug mitochondrial genomes which allowed them to be circularized. All mitochondria ranged between 13 and 16 kbps in size and contained the 13 protein coding genes found in previously described pentatomid mitochondria^69^, two rRNA genes and 22 tRNAs. Gene order was conserved across all genomes. COX1 genes were searched against the BOLD database confirming species identification for 9 samples, while in 6 cases for specimens collected in the Neotropics a certain match was not found, likely due to the species not being present in the database (See Table S4).

**Figure 2:**
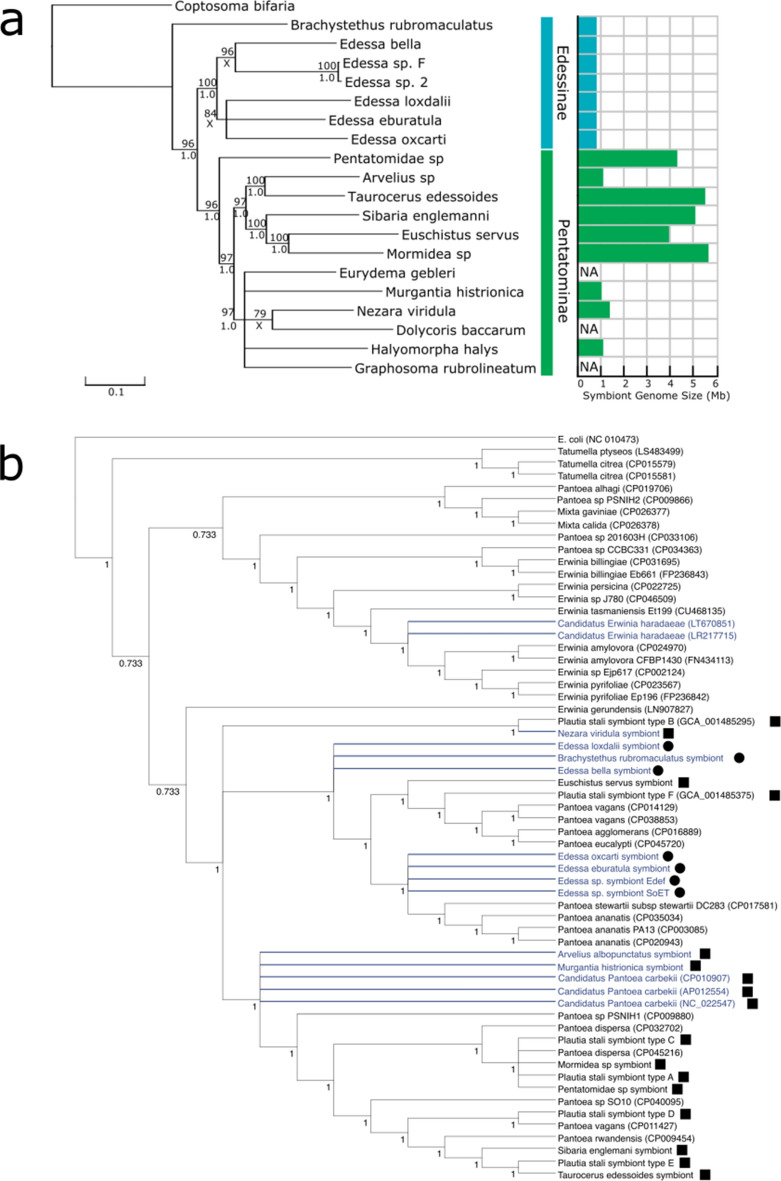
Host and symbiont relationships: (**a**) Stink bug mitochondrial tree with their respective symbiotic bacterial genome size. Nodes with lower than 60% bootstrap support were collapsed. Numbers above nodes represent bootstrap support while numbers underneath represent posterior probability from Bayesian Inference run. X indicates lower than 0.8 posterior probability. NA-genome size was not available. (**b**) Consensus cladogram of representative genomes of Erwiniaceae and stink bug symbionts. Individual FastTree reconstructions for the 10 longest protein sequences identified to be common for all taxa using reciprocal best hit Blast. *E. coli* was used as an outgroup. NCBI accession numbers for genomes are shown in parenthesis. Squares indicate a symbiont of a member of the Pentatominae while circles indicate symbionts of Edessinae. Numbers at nodes indicate Shimodaira-Hasegawa (SH)-like local support values. Taxa that were individually reconstructed are shown as polytomies in blue.

The reconstructed host mitochondrial tree showed good support for the genus *Edessa* (Fig. 2a, S1), containing the smaller symbiont genomes sequenced. The *Brachystethus* genus, also in the subfamily *Edessinae*, contains a symbiont of similar size, however the subfamily node was not well supported under this analysis (Fig. 2a). Additionally, despite similar sizes the genome of SoBr has several key differences from those of the SoE (See below). For the second subfamily, the *Pentatominae*, we see a large variation in symbiont genome sizes, ranging from moderately reduced (~ 1 Mb) to showing no signs of reduction (> 5 Mb).

### Placement of symbiotic strains within the Erwiniaceae

Traditional phylogenetic reconstruction approaches were not used because long branch attraction artifacts^60^ often confound accurate reconstruction of phylogenies of genome reduced species. We used a modified approach where separate trees were estimated for each genome reduced taxon and a consensus tree was created where all genomes were placed in the genus *Pantoea* and stink bug symbionts were shown to be paraphyletic within the genus (Fig. 2b). While these placements are tentative due to the lack of resolution on nodes with long branches, it provides evidence for the paraphyly of these symbionts.

### Branched chain amino acid biosynthesis pathway

**Figure 3:**
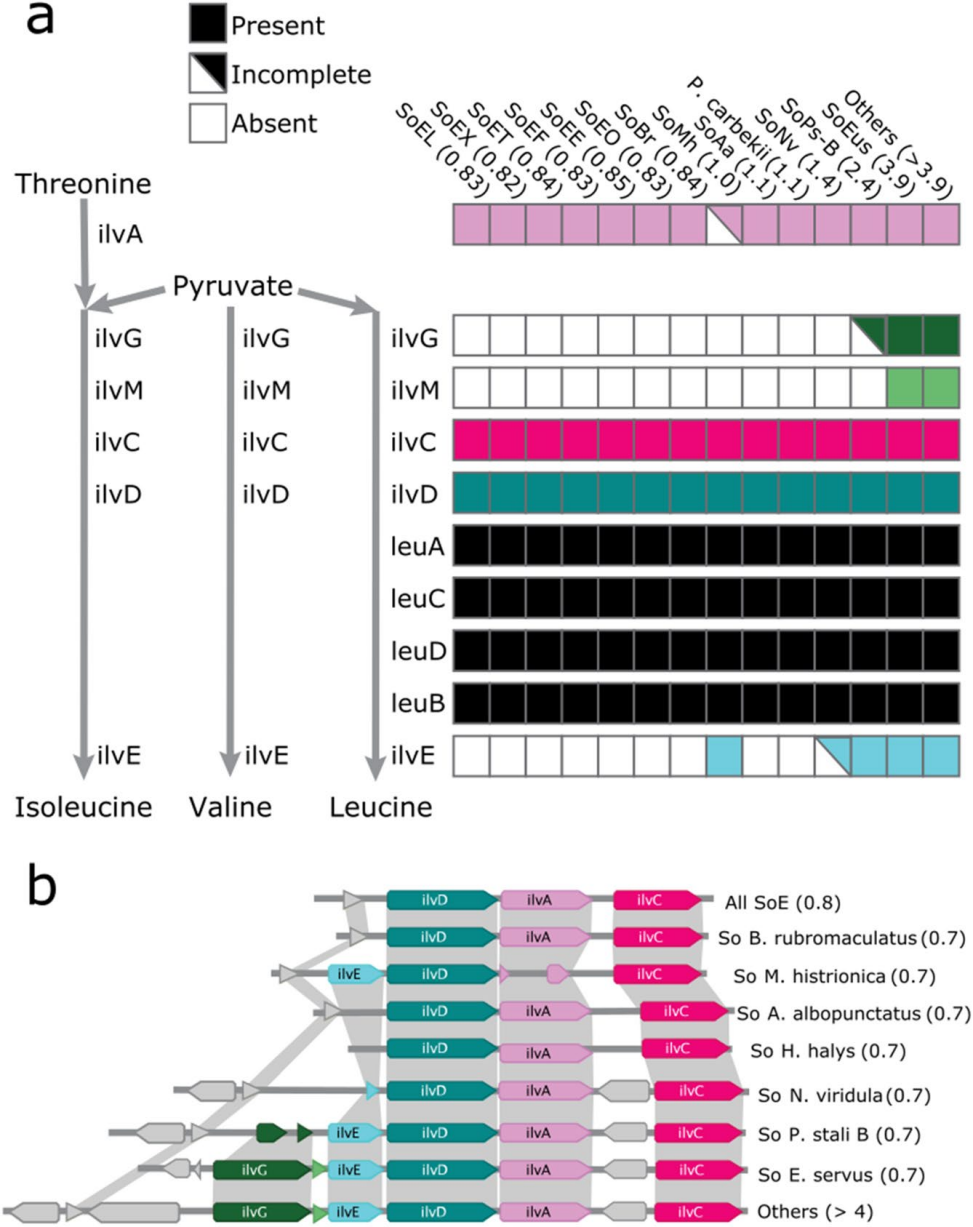
Presence of genes involved in branched chain amino acid biosynthesis pathway. The presence of genes in each genome according to the order of the pathway (**a**) and gene order and presence in the ilv operon region (**b**). Numbers in parentheses represent genome sizes in Mb. Colors are used to match genes in between panels for ease of visualization. Unlabeled grey pentagons denote genes not directly involved in the pathway.

The supplementation of essential amino acids not present in phytophagous pentatomid host diets is one of the likely advantages of these inheritable symbionts^18,70^. We identified the canonical biosynthetic pathways for these amino acids, and found all genomes contained full pathways for the biosynthesis of all essential amino acids with the exception of the branched chain amino acid biosynthesis pathway. This is in contrast with nonessential amino acid pathways in which several key enzymes are missing from some of the stink bug symbiont genomes (for a more detailed description see^36^. The only loss in an essential amino acid pathway is in the ilv operon. The ilv operon encoding most of the pathway^71^ is present in all genomes with the particularity that the *ilvE* gene which encodes the branched-chain-amino-acid aminotransferase BCAT, the enzyme responsible for the final step in the valine, isoleucine and leucine biosynthesis pathway is missing in all symbionts with a genome under 2 Mb, with the exception of SoMh (Fig. 3a). In the SoNv, *ilvE* gene is in the process of being pseudogenized, being present as a small ORF that does not include any of the protein’s catalytic sites but retains nucleotide and protein identity to the functional genes in other genomes (Fig. 3b). *ilvG* and *ilvM* are also missing from these genomes, which when present are located adjacent to *ilvE* and in SoPs-B *ilvG* is in a similar process of pseudogenization as *ilvE* in SoNv.

While *ilvE* was present in the genome of the SoMh, being the only genome under 2 Mb to retain this gene, it is also the only genome of those sampled to contain a pseudogenized *ilvA* gene, which is completely conserved in all others. *ilvA* encodes L-threonine deaminase, which catalyzes the first step of the synthesis of isoleucine from threonine which shares the remaining steps with the other branch chain amino acid synthesis pathways^72^.

### LPS and antigen biosynthesis gene loss during genome reduction

External cell wall components are responsible for protection of the cell in an outside environment but also are in direct contact with the host while in symbiosis. We compared the genes and pathways involved in some important and well conserved cell wall components: lipid A, peptidoglycan (PG), the O-antigen, and the enterobacterial common antigen (ECA). Lipid A is a precursor for the outer cell membrane lipopolysaccharide (LPS) present in Gram-negative bacteria including the Enterobacteria^73^. All genomes contained the necessary genes for the production of UDP-N-Acetyl-D-glucosamine (UDP-GlcNAc) *pgi* and the *glm* operon, which is a necessary precursor for both Lipid A and peptidoglycan^74,75^. The genes necessary for the production of peptidoglycan, *murABCDEFGIJ, ddl,* and *mraY* are also present in all genomes studied.

**Figure 4:**
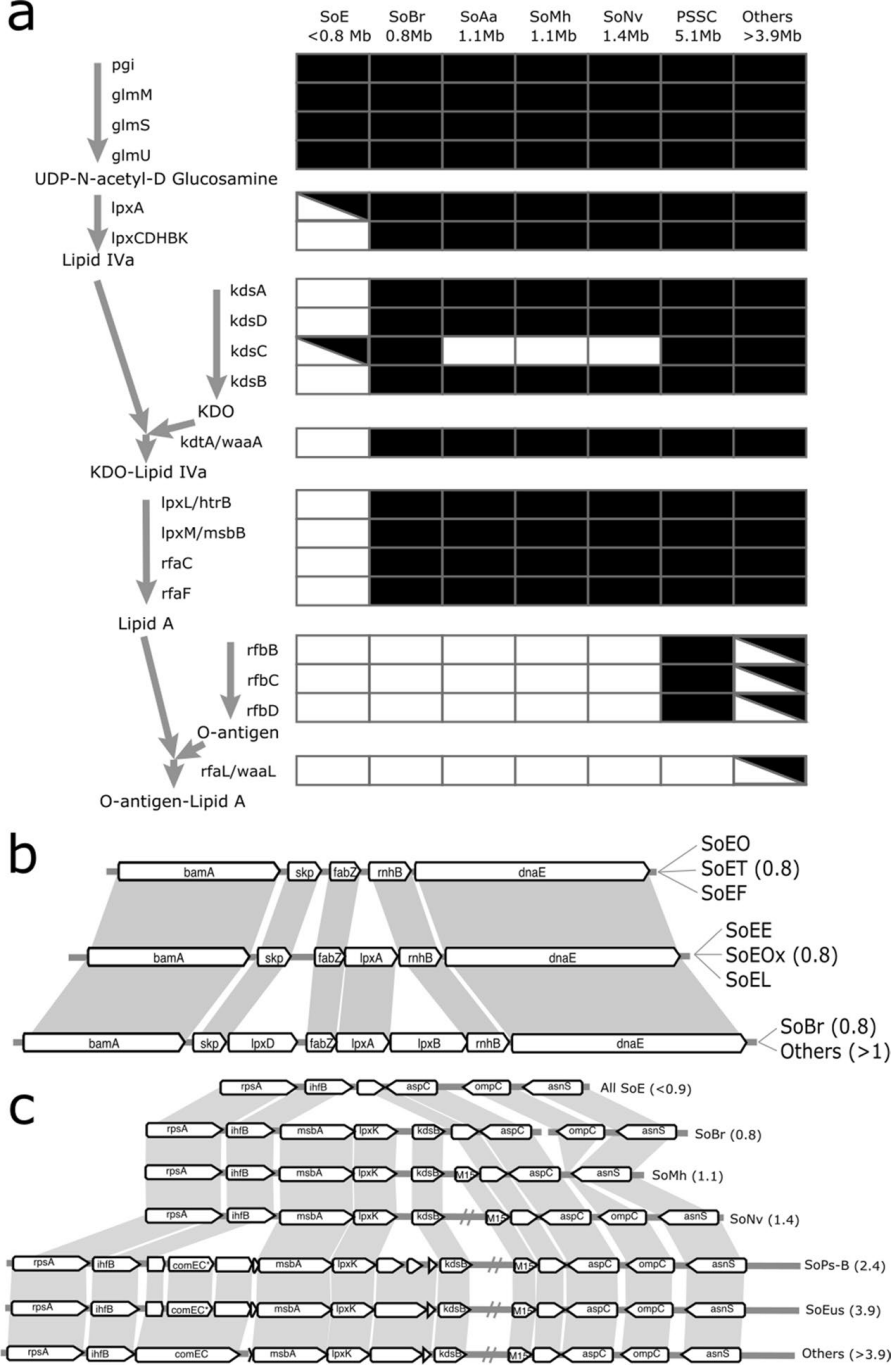
Genes involved in the production of Lipid A and attachment of O-antigen. (**a**) Intermediate metabolites are represented in bold and the presence or absence of a gene is represented in the table, with a crossed box indicating not present in all members. (**b**) Progressive elimination of lpxA, lpxB, and lpxD in the SoE. (**c**) Loss of lpxK kdsB, and adjacent genes. A double grey line indicates a region of approximately 15 kb containing several genes not shown here. Genes labeled ‘hypoth.’ or unlabeled indicate hypothetical proteins or interrupted CDS. Asterisk indicates a fragmented CDS. Numbers in parentheses correspond to genome sizes in Mb.

The canonical pathway encoding the production of lipid A from UDP-GlcNAc includes the genes *lpxACDHBK* (for the conversion to lipid IV_A_), *kdtA* (for the addition of KDO), *lpxLM* (conversion to KDO-Lipid A) and *rfaCFGPQYBOJ* (synonym *waa*) (addition of sugars to Lipid A). The pathway for the synthesis of lipid IV_A_ is conserved in all genomes larger than 1 Mb but lost in smaller ones, with the exception of *lpxA* which is found in some SoE and SoBr and *lpxB* which is found in SoBr (Fig. 4a,b). In some cases, these genes are lost without any change to the adjacent genes (such as the case of *lpxC*, see Figure S2) while in others, regions containing several adjacent genes are lost (such as the case of *lpxL*, Fig. 4c).

The genes *lpxD*, *lpxA*, and *lpxB*, are located syntenically in the *Pantoea* genomes along with *fabZ*, *rnhB*, and *dnaE* in a conserved pattern (Fig. 4b). This region is disrupted in the different genome reduced symbionts: The SoE lack *lpxB* and *lpxD*, and some (SoEO, SoEF) also lack *lpxA*. Despite this loss, the adjacent genes *fabZ* and *rnhB* are retained in these genomes. *lpxC* is found in all genomes between the *ftsZ* and *secA* genes, but it is lost in the SoE, where these two genes are adjacent to each other with no ORFs in the intergenic space. *lpxH* is also lost on all SoE along with the adjacent gene *ppiB*. However, genes flanking these two, *cysS* and *purE*, are conserved in the remaining genomes, the exception being *P. carbekii*, where *ppiB* and *lpxH* are retained but *purEK* are lost, which are part of the purine biosynthesis pathway. This loss is also present in SoEO. *lpxK* is also absent in all SoE genomes, in a gap where several genes are missing. In other genome reduced symbionts, *lpxH* is retained together with some adjacent genes that are absent in SoE. This region contains several gaps of multiple genes that are conserved in large genomes (Fig. 4c).

Adjacent to this region, we see evidence of pseudogenization of another gene, *comEC*, in the larger genomes that have begun the reduction process. In SoEus and SoPs-B, with genomes of 3.9 and 2.4 Mb respectively, *rpsA* and *ihfB* sit upstream of *comEC* while *msbA* and *lpxK* sit downstream. While in smaller genomes such as SoNv only a small intergenic region between *ihfB* and *msbA* remains, in SoEus and SoPs-B the region is roughly the same length as in unreduced genomes and contains several small, interrupted ORFs, some similar to the full protein, but likely not functional.

Another requirement for the production of lipid A are the *kdsABCD* genes in charge of the production of KDO. The first gene in the pathway, *kdsC* is the most conserved, present in all genomes except some SoE (SoEL & SoEE retain it). In SoEus, *kdsC* is split into two ORFs, indicating a possible pseudogenization occurring, and it is unsure if either of these have catalytic function. These results together show how the SoE have a complete or almost complete loss of the lipid A biosynthesis pathway, while others, including the similarly sized symbiont SoBr, conserve all genes.

The O-antigen consists of an oligosaccharide that can be attached to Lipid A to produce a mature LPS. Several glycosyltransferases can be involved in this process, in the case of *Pantoea* the *rfbBCD* genes encode enzymes involved in the production of dTDP-beta-L-rhamnose, a monomer of their O-antigen. This operon is absent in all genomes under 2 Mb, as well as some of the larger genomes, including those of the non-associated *Pantoea* species. The Enterobacterial common antigen, or ECA, is another antigen that can be found in the periplasmic space in a cyclic form, attached to PG or attached to LPS. The genes for its production *wecABDEFG*, and *rffG*, as well as the gene in charge of producing the cyclic form *wzzE* are lost in all genomes smaller than SoPs-B. *rfaL*, which binds both the O-antigen and the ECA to the LPS, and *wzxE* and *wzyE* which are in charge of the export of both antigens^76,77^ are also lost in smaller genomes.

## Discussion

### Different stages of reduction among pentatomid stink bugs

There is a large variation in genome sizes of inhabitants of the M4 region of stink bugs in the family Pentatomidae. The placement of these symbionts is in the genus *Pantoea* consistent with previously discovered pentatomid symbionts^30,35,68,78^. The true relationships between bacteria with reduced genomes can be incredibly difficult to ascertain because accurate phylogenetic reconstructions are confounded by elevated mutation rates that can result in artifactual long branch attraction. However, the radically different genome size, different placement of taxa in the absence of long branches, as well as considerable differences in the gene order between these different organisms^36^ are all indications of separate association events at different times. The SoE have almost identical genome size and gene order amongst each other, while being considerably different from that of *P. carbekii* indicating these to be separately evolving groups. However, gene order, size, and independent phylogenetic placement between *P. carbekii* and SoMh or SoAa are not similar enough to ascertain that they belong to the same clade and not different enough to claim the contrary. Therefore, while largely different genome sizes are likely an indication of separate clades, similar genome sizes should not be taken as evidence of the same clade and comparisons must be cautious. For example, SoBr is of a similar genome size to the SoE and while *B. rubromaculatus* is the only other member of the *Edessinae* that is not in the genus *Edessa*, there are considerable differences between them, most prominently the latter retaining the full pathway for synthesis of lipid A. Gene order in SoBr is similar to that of the SoE, but to a lesser extent as within the genus, in which gene order is near identical.

While some of these genomes are considerably small for extracellular bacteria, undoubtedly at a late stage of genome reduction, examples such as the symbiont of *P. stali*-B and the symbiont of *N. viridula* allow us to see a glimpse of the intermediate stage of this process. Additionally, the larger genomes of symbionts, while retaining a total genome size similar to their non-host associated relatives, show several characteristics of an early stage genome reduction such as a considerable number of pseudogenes and a decrease in the coding density of the genome. This likely reflects a recent symbiont replacement event in which the host associated with a new clade, as SoEus is shown to be closely associated with *P. vagans* and *P. agglomerans* while SoPs-A and SoPt are more closely associated with *P. dispersa* (Fig. 2b). For SoPs-A, a shift in geography may have facilitated this replacement as different populations in separate islands contain different associates, including a more genome reduced SoPs-B^27^. The variation in genome sizes indicates that the replacement process can be relatively common and actively occurs in species of this family^35^. Given the extracellular nature of these bacteria and the host transmission and acquisition system, it is not difficult for other bacteria to invade. However, the high symbiont titers of genome reduced species show colonization must be favored for closer associates. In the case that genome reduction proceeds to an irreversible deletion within the symbiont that affects its utility to the host, it may more advantageous for the host to replace it^12,79^ with a large genome-bearing bacterium and start the process anew.

A wide variety of genome reduced insect symbionts has been described, covering the full range of genome sizes down to near organelle levels and with great diversity in the association to their host. Several of these symbionts have been hypothesized to be in a transitional state towards a stable symbiosis^80^, while others have severely reduced functions as in the case of intracellular bacteria with small genomes that are approaching organelle status^81^. Many of these cases require the host evolution of traits such as methods for intracellular vertical transmission^82^, a second symbiont’s complementation^83^, or even horizontal gene transfer of symbiont genes to the host^84^. However, most of these cases allow a limited view on an individual symbiosis without understanding the gradual steps required to achieve it, allowing us only to understand the process of symbiosis by comparing sometimes distant hosts with different organs, lifestyles, and diets. Here, we show how within a single family of stink bugs with little host change of the symbiotic organ we can evidence a range of steps in the development of the symbiosis from the bacterial perspective due to repeated establishment of the symbiosis.

While most of the extremely reduced genomes of symbionts are from intracellular symbionts, which are carefully protected within host cells, extracellular symbionts have additional constraints that likely impede further genome reduction. However, these can be overcome if there is significant investment from the host on structures that guarantee housing and transmission of its bacterium^85–87^. Stink bug symbionts are housed in separate gut compartments (or crypts) developed with a complex morphogenetic process from birth to adulthood^26^ and they are externally transmitted^17,23,37^, although it is unclear to what degree these symbionts are impacted by abiotic conditions outside of the host (i.e. temperature, dessication, UV light) given that they are often ensconced in maternal secretions. Some further strategies have been developed in other stink bug relatives such as a symbiont capsule in Plataspidae^88^ or transmission jelly in the Urostylididae^20^ which may have enhanced genome size reduction, but SoE species have similarly-reduced genomes but are not transmitted in either a capsule or jelly^34,36^. Additionally, some genome reduction may co-occur as a relationship with an amenable domiciling host emerges, as observed in deep sea anglerfish-associated luminescent symbionts with genomes that have undergone ‘extreme reduction’ that is likely impacted by association with anglerfish but independent of obligate intracellular incarceration^89,90^.

Cases where change is required in the physiology of the host to accommodate a symbiont require longer evolutionary time due to the difference in generation time and population size^1^. These traits can vary considerably, such as with the different symbiotic organs of Lygaeid bugs^91–93^, which makes it unlikely for the exact same structure to appear convergently. Thus, comparison of genome reduction for these cases can only be done with distantly related taxa, if at all. In the case of stink bugs, the midgut crypts are common to most of the Pentatomoidea (except in cases where they were subsequently lost such as for carnivorous groups or otherwise modified)^17–19,94^ and the nymphal probing behavior is also common across the superfamily^23,95^. Since host traits that enable the symbiosis are almost identical across the group, yet the symbionts appear at radically different stages of association, this system is invaluable for understanding the effects of varying constraints on genome composition.

### Common genes after genome reduction

The core genome obtained for the unreduced genomes consists of 2450 genes, which includes between 46.6% and 61.4% of the total genes in the genome. Our estimate is slightly larger than previous core genome analyses of the *Pantoea*^96,97^ which estimate them at between 38.8–56% and 30% of the total genes, respectively. However, previous analyses included more distant strains of *Pantoea* and more samples for some of species which could contribute to their smaller core genome. When including all symbionts down to the SoE, the number of shared genes is reduced to 450 genes, which constitutes between 47.7% and 62% of the genes in the smaller genomes. This estimate is likely low given that these methods use sequence similarity to identify orthologous genes which can result in false negatives because the high mutation rate of genome reduced bacteria significantly lowers the sequence identity of orthologous proteins. We observed some cases where a group of shared genes was incorrectly counted as two non-overlapping groups due to insufficient sequence identity, even though annotations and genome position for the genes was identical. This is a considerable problem for organisms with such high mutation rates and should be carefully considered in comparative genomic frameworks.

### Amino acid pathway loss

*ilvE* is the only gene in the valine, isoleucine, and leucine biosynthesis pathway lost between the core genome of *Pantoea* and the set of shared genes for all genomes. It encodes BCAT which catalyzes the last step for the production of valine, leucine and isoleucine. This gene is missing from multiple other nutritional symbionts^5,98–100^. In *Buchnera* it has been shown the host aphid upregulates its own BCAT in its bacteriocytes^3^ completing the pathway. BCAT is also present in the brown marmorated stink bug genome (LOC106691811) indicating stink bugs may also be completing this pathway in the symbiosis. The loss of *ilvE* in these cases also comes with the loss of *ilvG* and *ilvM*, which encode the two subunits of acetolactate synthase, which catalyzes the first step of the branched-chain amino acid biosynthesis pathway ^101^. However, there are three isozymes with this function in other Enterobacteria such as *E. coli*, among them IlvIH, encoded by *ilvI* and *ilvH*^102^, which is found in all genomes including those of the SoE. The exception to the loss of *ilvE* is SoMh, which retains a full copy of *ilvE*. However, it is unique among the stink bug symbionts in the loss of *ilvA*, a gene encoding L-threonine dehydratase [E.C:4.3.1.19] which is required for a previous step in the biosynthesis of isoleucine (Fig. 4a). This loss is also present in *Buchnera* and *Wigglesworthia*, and in aphids can be replaced with the host enzyme TcdB which is expressed and marginally upregulated in the bacteriocytes^3^. A similar protein is found in the genome of the closest pentatomid genome (*H. halys*, LOC106681826). This could be evidence of an alternative evolution of a shared pathway between host and symbiont, regulating exclusively the isoleucine pathway instead of all three branched-chain amino acids. This complementation is found in multiple genome reduced symbionts and their hosts, including other pathways such as tyrosine supplementation in weevils^103^. The loss of a vital step of the biosynthesis pathway for the symbiosis is likely beneficial for the host as it facilitates increased control over the production of the required nutrient and the growth of the symbiotic bacteria^104^.

The genomic region containing the previously mentioned ilv genes is a clear example of progressive gene decay: SoPs-B contains an identical gene order to large genome symbionts but has lost *ilvM* and *ilvG* is in the process of pseudogenization, the next smaller genome SoNv has lost *ilvG* altogether and *ilvE* is being pseudogenized, and finally in SoAa and *P. carbekii ilvE* is lost altogether. Since IlvGM is redundant it is likely one of the fastest genes to disappear, and *ilvE* or *ilvA* disappear in the next stage.

### Cell membrane component loss during symbiosis

Lipopolysaccharides (LPS) are the main component of the exterior cell membrane of Gram-negative bacteria acting as a protective barrier for the cell. It is composed of Lipid A, which binds to the cell membrane, a core oligosaccharide, and an external polysaccharide chain sometimes referred to as the O-antigen. There are both conserved regions as well as considerable variation in the composition of LPS, and particularly the O-antigen region is a target of host immune responses^105^. We identified two major changes in the biosynthetic capability of stink bug symbionts with regards to LPS: the first being the loss of addition of O-antigen and ECA and the second being the complete loss of LPS. We found that the genes responsible for the production and attachment of the ECA and O-antigens to lipid A are absent in stink bug symbionts with reduced genomes which would render them unable to produce smooth-type (antigen containing) LPS. In other species of the genus *Pantoea* and symbionts with regular genome size the rfb operon can be complete (*P. rwandensis*, SoEus, SoPs-A, SoSe and SoPd) or incomplete (*P. ananatis*, SoMo, SoTe, and other SoPs) which indicates the addition of the O-antigen is not essential for the survival of the bacteria. *E. coli* mutants are able to survive without the addition of O-antigen, however, their membrane is considerably more permeable making the it hypersusceptible^106^. The ECA behaves similarly, while it is not essential for the survival of strains in culture, outer membrane permeability is considerably reduced^76^.

In the *Burkholderia* and bean bug *Riptortus pedestris* symbiosis, symbiont *Burkholderia* cells produce LPS with O-antigen when grown in culture media but the O-antigen is absent in symbiotic cells. Additionally, symbiotic *Burkholderia* do not induce host antimicrobial peptides (AMPs), and the expression of host AMPs in the M4 region is lower than the basal expression in the fat bodies. It has been shown that cells without the O-antigen are much more susceptible to cell lysis and host immune responses. However, this downregulation of AMPs in the symbiotic organ likely allows the survival of a weakened symbiotic cell^107^. It is likely that as in the bean bug-*Burkholderia* symbiosis, the weakened membrane due to the loss of O-antigen evidenced in the stink bug symbiont genomes is compensated by the host protection and/or changes in its immune reaction. The loss of these genes in stink bug symbionts is likely due to increased pressure to prevent the activation of the host’s immune system or as a different adaptation to the host environment^108^. Since these symbionts need to travel through the host gut during its first instar in order to colonize, as well as proliferate throughout the host’s development, the loss of this antigen is likely helpful if not necessary for the consistent establishment of symbiont populations, however, a there is a trade-off involved of increased cell permeability which may be related to the inability of genome reduced symbionts to grow in vitro. Additionally, as with *Burkholderia*, unreduced genome bacteria may be able to stop production of these antigens when in a symbiotic state.

Furthermore, we found that all SoE lacked the complete pathway to produce lipid A (with the exception of the gene *lpxA* encoding UDP-GlcNAc acyltransferase which catalyzes the first step in this pathway, which was present in SoEL, SoEE, and SoOX). Under normal circumstances the disruption of lipid A is fatal for most gram negative bacteria, with few exceptions^109,110^. While the SoE lacked the ability to produce lipid A, the production of UDP-GlcNAc remained intact in all genomes. The only other pathway that this metabolite has been associated with is in the biosynthesis of peptidoglycan. All genomes of stink bug symbionts, including *I. capsulata* and *T. gelatinosa*, contained all genes necessary for the production of peptidoglycan, which is found in the periplasmic space. Only the most reduced genome symbionts in other systems are able to lose production of peptidoglycan, and these are restricted to intracellular symbionts and those that can rely on host production of peptidoglycan through horizontally transferred genes^83,111^. Given that stink bug symbionts are extracellular bacteria, we hypothesize that maintenance of LPS in the outer membrane is not necessary for an extracellular symbiosis but peptidoglycan production in the periplasmic space must be conserved likely for cell wall integrity. This hypothesis could be tested in systems such as the extracellular *Stammera* – tortoise leaf beetle symbiosis, where some symbionts retain some, all, or almost none of the genes required for peptidoglycan biosynthesis^86^.

## Conclusion

Genome reduction has been widely studied across insect symbionts, yet comparative genomic methods studying convergence in genome evolution have been limited to either too distant or too similar instances of symbiosis. Here we show how the extracellular symbionts of stink bugs can be used as a system of similar and convergent instances of genome reduction, as well as covering the range of symbiosis from free living bacteria to highly specialized symbionts. We identify convergence in gene loss in multiple pathways associated with symbiosis and different stages of loss including partial gene losses and ongoing pseudogenization. We identify a convergence in the loss of a single gene in branched chain amino acid biosynthesis, with one example displaying the loss of a different step in the pathway (*ilvA* as opposed to *ilvE*) found in other nutritional symbionts, as well as the selective loss of genes involved in the production and attachment of antigens to the cell wall which likely influences interactions with the host. Further study of the diversity and evolution of these symbionts will likely elucidate key factors in the process of genome reduction.

## Supplementary Information

Supplementary information.

## Data Availability

These Whole Genome Shotgun projects have been deposited at GenBank under the NCBI accessions SZZU00000000, VOQV00000000, VOQW00000000, SZZT00000000, SZZY00000000, VOQX00000000, SZZX00000000, SZZW00000000, VOQY00000000, SZZV00000000, PDKT00000000, PDKU00000000, PDKR00000000, PDKS00000000, SZZZ00000000.

## Data deposition

Stink bug mitochondrial genomes have been deposited at GenBank under the NCBI accession numbers MN783643-MN783657 and bacterial genomes have been deposited under BioProject PRJNA413893.
