# Supplementary material for: Multiple concurrent and convergent stages of genome reduction in bacterial symbionts across a stink bug family
Source: Sci Rep. 2021 Apr 8;11:7731. doi: 10.1038/s41598-021-86574-8 (PMC8032781; doi:10.1038/s41598-021-86574-8)
Supplement: Supplementary file 1 — Supplementary information. [file 41598_2021_86574_MOESM1_ESM.docx]

SUPPLEMENTARY MATERIALS

# Title

Multiple concurrent and convergent stages of genome reduction in bacterial symbionts across a stink bug family

# Authors

Alejandro Otero-Bravo^1,2^ and Zakee L. Sabree^1*^

^1^Department of Evolution, Ecology and Organismal Biology, Ohio State University, Columbus, Ohio, 43210, USA

^2^Nationwide Children’s Hospital, Columbus, Ohio, 43205, USA

^*^Corresponding Author, 318 W. 12^th^ Avenue, Columbus, Ohio, 43210. 614-688-1590. [sabree.8@osu.edu](mailto:sabree.8@osu.edu)


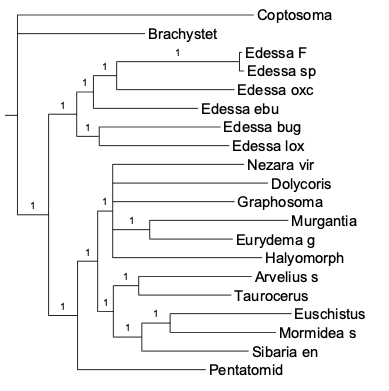


Figure S1. Bayesian inference consensus tree for host mitochondrial genomes.


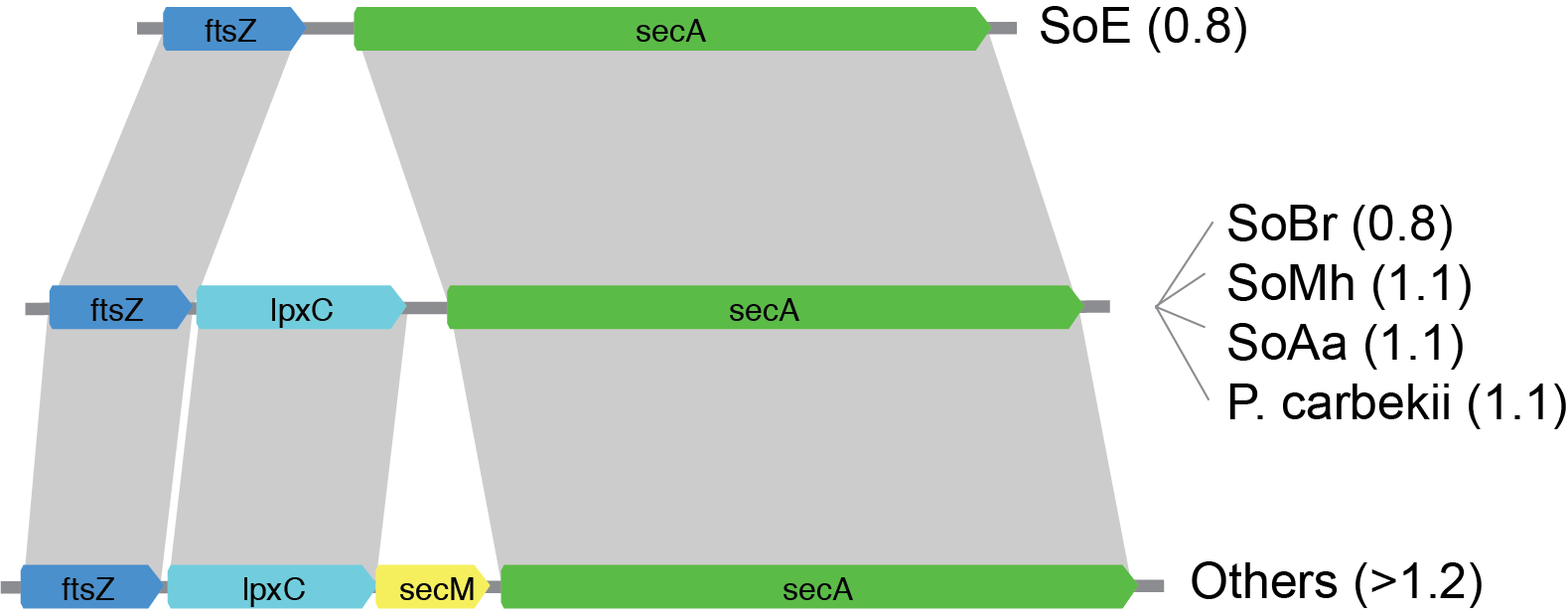


Figure S2. lpxC loss.

| Table S1. Accession numbers used for Pangenome analyses | |
| --- | --- |
| Bacteria | ACC |
| Arvelius_albopunctatus_symbiont | SZZU00000000 |
| Brachystethus_symbiont | VOQV00000000 |
| Edessa_F_symbiont | VOQW00000000 |
| Edessa_oxcarti_symbiont | SZZT00000000 |
| Euschistus_servus_symbiont | SZZY00000000 |
| Mormidea_symbiont | VOQX00000000 |
| Murgantia_histrionica_symbiont | SZZX00000000 |
| Nezara_viridula_symbiont | SZZW00000000 |
| Plautia stali symbiont A | BBNZ00000000 |
| Plautia stali symbiont B | BBOA00000000 |
| Plautia stali symbiont C | BBOB00000000 |
| Plautia stali symbiont D | BBOC00000000 |
| Plautia stali symbiont E | BBOD00000000 |
| Plautia stali symbiont F | BBOE00000000 |
| Pantoea agglomerans | GCA_001709315.1 |
| Pantoea ananatis | GCA_000025405.2 |
| Candidatus Pantoea carbekii | GCA_000971765.1 |
| Pentatomidae_sp_symbiont | VOQY00000000 |
| Pantoea rwandensis | GCA_000759475.1 |
| Pantoea stewartii | GCA_002082215.1 |
| Sibaria_englemani_symbiont | SZZV00000000 |
| Edessa eburatula symbiont | PDKT00000000 |
| Edessa loxdalii symbiont | PDKU00000000 |
| Edessa sp nov 1 symbiont | PDKR00000000 |
| Edessa sp. 2 symbiont | PDKS00000000 |
| Taurocerus_edessoides | SZZZ00000000 |

| Table S2. Accession numbers for members of the Erwiniaceae used for placement with FastTree. | |
| --- | --- |
| Label | **Accession** |
| Pantoea sp PSNIH2 | CP009866 |
| Mixta gaviniae | CP026377 |
| Pantoea rwandensis | CP009454 |
| Pantoea vagans | CP011427 |
| Pantoea sp SO10 | CP040095 |
| Pantoea dispersa | CP045216 |
| Pantoea dispersa | CP032702 |
| Pantoea sp PSNIH1 | CP009880 |
| Candidatus Erwinia haradaeae | LT670851 |
| Candidatus Erwinia haradaeae | LR217715 |
| Candidatus Pantoea carbekii | AP012554 |
| Candidatus Pantoea carbekii | NC_022547 |
| Pantoea ananatis | CP020943 |
| Pantoea ananatis PA13 | CP003085 |
| Pantoea ananatis | CP035034 |
| Pantoea stewartii subsp stewartii DC283 | CP017581 |
| Pantoea eucalypti | CP045720 |
| Pantoea agglomerans | CP016889 |
| Pantoea vagans | CP038853 |
| Pantoea vagans | CP014129 |
| Plautia stali symbiont type F | GCA_001485375 |
| Plautia stali symbiont type B | GCA_001485295 |
| Erwinia gerundensis | LN907827 |
| Erwinia pyrifoliae Ep196 | FP236842 |
| Erwinia pyrifoliae | CP023567 |
| Erwinia sp Ejp617 | CP002124 |
| Erwinia amylovora CFBP1430 | FN434113 |
| Erwinia amylovora | CP024970 |
| Erwinia tasmaniensis Et199 | CU468135 |
| Erwinia sp J780 | CP046509 |
| Erwinia persicina | CP022725 |
| Erwinia billingiae Eb661 | FP236843 |
| Erwinia billingiae | CP031695 |
| Pantoea sp CCBC331 | CP034363 |
| Pantoea sp 201603H | CP033106 |
| Mixta calida | CP026378 |
| Pantoea alhagi | CP019706 |
| Tatumella citrea | CP015581 |
| Tatumella citrea | CP015579 |
| Tatumella ptyseos | LS483499 |
| E. coli | NC 010473 |
| Candidatus Pantoea carbekii | CP010907 |

| Table S3. SILVA identification | | | | | |
| --- | --- | --- | --- | --- | --- |
| Species | SILVA score | identity | Order | Family | Genus |
| Arvelius albopunctatus symbiont | 0.937867 | 94.3005 | Enterobacterales | Erwiniaceae |  |
| Brachystethus rubromaculatus symbiont | 0.926469 | 93.391 | Enterobacterales |  |  |
| Edessa_F symbiont | 0.910574 | 92.5432 | Enterobacterales |  |  |
| Edessa oxcarti symbiont | 0.918434 | 91.9216 | Enterobacterales |  |  |
| Euschistus servus symbiont | 0.995991 | 99.6601 | Enterobacterales | Erwiniaceae | Pantoea |
| Mormidea sp symbiont | 0.995271 | 100 | Enterobacterales | Erwiniaceae | Pantoea |
| Murgantia histrionica symbiont | 0.946696 | 93.8472 | Enterobacterales | Erwiniaceae | Pantoea |
| Nezara viridula symbiont | 0.999383 | 100 | Enterobacterales | Erwiniaceae | Pantoea |
| Pentatomidae sp symbiont | 0.994315 | 99.6288 | Enterobacterales | Erwiniaceae | Pantoea |
| Sibaria englemani symbiont | 0.999432 | 99.3662 | Enterobacterales | Erwiniaceae | Pantoea |
| Taurocerus sp symbiont | 0.999421 | 98.9699 | Enterobacterales | Erwiniaceae | Pantoea |

| Table S4. COX1 results from BOLD ID. | | | |
| --- | --- | --- | --- |
| ACC | ID | BOLD ID | Note |
| MN783643 | Arvelius albopunctatus | - |  |
| MN783644 | Brachystethus rubromaculatus | Brachystethus rubromaculatus |  |
| MN783645 | Edessa bella/sp nov 1 | Edessa bugabensis |  |
| MN783646 | Edessa eburatula | Edessa eburatula | Other similar sequences found |
| MN783647 | Edessa F. | - | Closest match to Edessa jugata (98.3%) |
| MN783648 | Edessa loxdalii | - |  |
| MN783649 | Edessa oxcarti | - |  |
| MN783650 | Edessa sp. CR2 | - | Closest match to Edessa jugata (98.15%) |
| MN783651 | Euschistus servus | Euschistus servus | Other similar sequences found |
| MN783652 | Mormidea sp. | Mormidea sp. | Match to M. collaris (99.07%) and M. ypsilon (98.92%) |
| MN783653 | Murgantia histrionica | Murgantia histrionica |  |
| MN783654 | Nezara viridula | Nezara viridula |  |
| MN783655 | Pentatomidae sp. | - |  |
| MN783656 | Sibaria englemanni | Sibaria englemanni |  |
| MN783657 | Taurocerus edessoides | Taurocerus edessoides |  |
